# Synthesis of a Cu^2+^-Selective Probe Derived from Rhodamine and Its Application in Cell Imaging

**DOI:** 10.3390/s141121375

**Published:** 2014-11-12

**Authors:** Chunwei Yu, Yingying Wen, Jun Zhang

**Affiliations:** Laboratory of Environmental Monitoring, School of Tropical and Laboratory Medicine, Hainan Medical University, Haikou 571199, China; E-Mails: yucw_1979@sina.com (C.Y.); wyy_418@163.com (Y.W.)

**Keywords:** rhodamine derivative, fluorescence enhancement, fluorescence imaging

## Abstract

A new fluorescent probe P based on rhodamine for Cu^2+^ was synthesized and characterized. The new probe P showed high selectivity to Cu^2+^ over other tested metal ions. With optimal conditions, the proposed probe P worked in a wide linear range of 1.0 × 10^−6^−1.0 × 10^−5^ M with a detection limit of 3.3 × 10^−7^ M Cu^2+^ in ethanol-water solution (9:1, v:v, 20 mM HEPES, pH 7.0). Furthermore, it has been used for imaging of Cu^2+^ in living cells with satisfying results.

## 1. Introduction

The selective detection of chemical species upon molecular recognition is of great significance in the host environment, particularly when the guest is ionic. Since fluoroionophores can provide chemical information on the ion concentrations, they are important subjects in metal ion analysis [1–5].

Among the metal ions, Cu^2+^ is one of the important targets, because it exhibits toxicity under overloading conditions, which can causes neurodegenerative diseases [6]. Thus, it is necessary to trace the concentration of Cu^2+^ *in vitro* and *in vivo*. Even though considerable efforts have been devoted to developing fluorescent probes for Cu^2+^ over the last few decades [7–9], it is still of great importance to develop new Cu^2+^-selective probes.

**Scheme 1.**
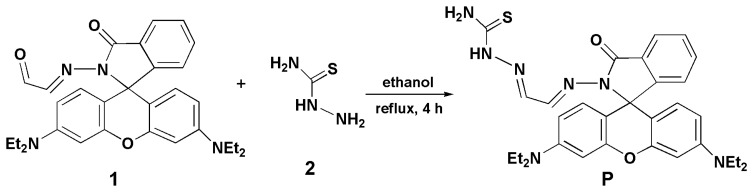
The synthesis route of probe P.

Owing to their excellent fluorescence properties, rhodamine dyes have been used extensively for conjugation with biomolecules [1,4,7]. Many rhodamine B derivatives have been used as fluorescent chemosensors for the detection of different metal ions [1,10,11]. Inspired by these research works, we report here a new fluorescent probe P based on a rhodamine B derivative for Cu^2+^ (Scheme 1). It showed a reversible “turn-on” fluorescent response for Cu^2+^ in aqueous solution with remarkably high sensitivity and selectivity. Moreover, it has been demonstrated that P can be used as a fluorescent probe for monitoring Cu^2+^ in living cells.

## 2. Experimental Section

### 2.1. Reagents and Instruments

All reagents and solvents are of analytical grade and used without further purification.

Nuclear magnetic resonance (NMR) spectra were measured with a Brucker AV 400 instrument, and chemical shifts were given in ppm from tetramethylsilane (TMS). Mass spectra (MS) were recorded on a Thermo TSQ Quantum Access Agilent 1100. Fluorescence emission spectra were conducted on a Hitachi 4600 spectrofluorometer. UV-Vis spectra were obtained on a Hitachi U-2910 spectrophotometer. Fluorescence imaging was performed by confocal fluorescence microscopy on an Olympus FluoView Fv1000 laser scanning microscope. pH was conducted with a pH-meter PBS-3C.

### 2.2. Synthesis of Compound P

Compound 1 [7] (1.0 mmol) and 2 (1.0 mmol) were stirred in ethanol (30 mL) at 80 °C for 4 h. After the reaction was finished, the precipitate so obtained was filtered and purified with silica gel column chromatography (Petroleum ether/acetic ether = 5:1, v/v) to afford P as a yellow solid. Yields: 67.2%. MS (ES+) m/z: 570.26 (M + H)^+^. ^1^H NMR (δ ppm, *d_6_*-DMSO): 11.58 (s, 1H), 8.62 (d, 1H, *J* = 8.28), 8.32 (s, 1H), 7.88 (d, 2H, *J* = 7.58), 7.58 (b, 2H), 7.54 (t, 1H, *J* = 7.22), 7.02 (d, 1H, *J* = 7.40), 6.42 (d, 3H, *J* = 8.68), 6.34 (d, 3H, *J* = 8.68), 3.33 (m, 8H, *J* = 7.42), 1.08 (t, 12H, *J* = 6.78). ^13^C NMR (δ ppm, *d_6_*-DMSO): 179.06, 165.27, 153.24, 152.69, 149.39, 147.19, 142.34, 135.17, 129.68, 128.52, 128.31, 124.57, 124.12, 109.00, 105.89, 98.39, 66.48, 44.56, 13.34 (Figures S1, Figures S2 and Figures S3).

### 2.3. General Spectroscopic Methods

Metal ions and probe P were dissolved in deionized water and DMSO to obtain 1.0 mM stock solutions, respectively. Before spectroscopic measurements, the working solution was freshly prepared by diluting the high concentration stock solution to the corresponding solution. For all of the measurements, excitation and emission slit widths were 10 nm, and the excitation wavelength was 520 nm.

### 2.4. Cell Incubation and Imaging

HepG2 cells placed on coverslips were washed with phosphate-buffered saline (PBS), followed by incubating with 1 μM of CuCl_2_ (in PBS) for 30 min at 37 °C, and then washed with PBS three times. After incubating with 10 μM of probe P for 30 min at 37 °C, the cells were washed with PBS three times again. Fluorescence imaging of intracellular Cu^2+^ in HepG2 cells was conducted by using a confocal fluorescence microscopy on an Olympus FluoView Fv1000 laser scanning microscope.

## 3. Results and Discussion

### 3.1. Effect of pH on P and P with Cu^2+^

**Figure 1.**
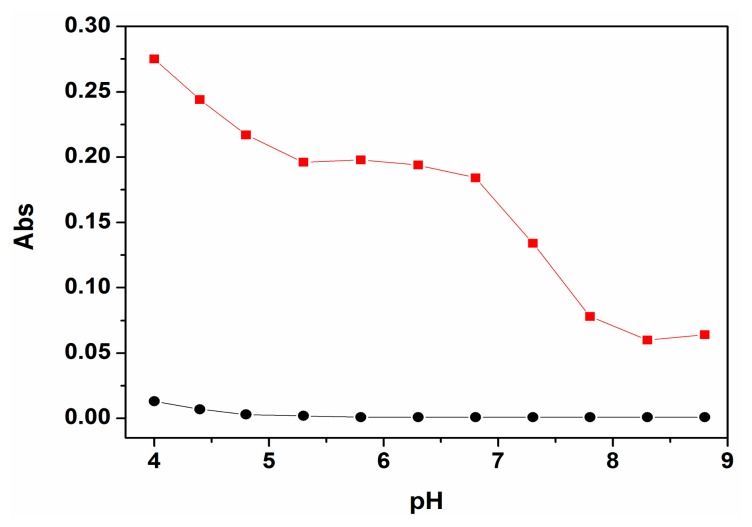
pH-dependence of P (10 μM) (•) and P (10 μM) plus Cu^2+^ (100 μM) (▪) in HEPES buffers as a function of different pH values in ethanol-water solution (9:1, v:v, 20 mM HEPES). The pH was modulated by adding 1.0 M HCl or 1.0 M NaOH in HEPES buffers.

In order to investigate a suitable pH working range of P for the sensing of Cu^2+^, a pH titration experiment was performed firstly (Figure 1). The results showed that the absorption of the free probe P can be negligible under a pH range from 4 to 9. After the addition of Cu^2+^, the absorption of probe P at 560 nm rapidly increased to a maximum value. The results showed that the probe P can be worked within a wide pH range of 5.3–7.0. As the pH of a natural water body is near neutral, therefore, further UV-Vis and fluorescent studies were carried out in ethanol-water solution (9:1, v:v, 20 mM HEPES, pH 7.0).

### 3.2. UV-Vis Spectral Response of P

**Figure 2.**
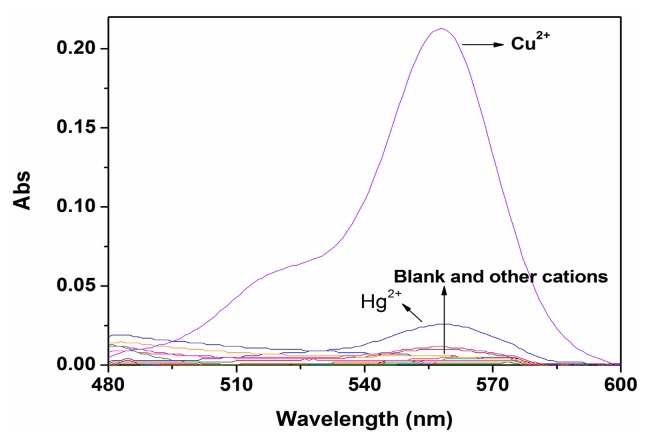
UV-Vis spectra of P (10 μM) in ethanol-water solution (9:1, v:v, 20 mM HEPES, pH 7.0) upon addition of different metal ions (100 μM).

As expected, probe P alone was colorless and scarcely showed absorption in the 500–600 nm region in ethanol-water solution (9:1, v:v, 20 mM HEPES, pH 7.0). However, upon addition of Cu^2+^, an intense absorption band centered at 560 nm appeared, presumably because of the chelation of Cu^2+^ with the nitrogen atom of the amide group of P, which resulted in the formation of the open-ring form of rhodamine B. At the same time, other related metal ions (K^+^, Na^+^, Ca^2+^, Mg^2+^, Zn^2+^, Pb^2+^ Co^2+^, Cd^2+^, Cr^3+^, Ni^2+^, Hg^2+^, Ag^+^, Fe^3+^ and Al^3+^) did not show any obvious absorption under similar conditions (Figure 2).

### 3.3. Fluorescence Spectral Response of P

**Figure 3.**
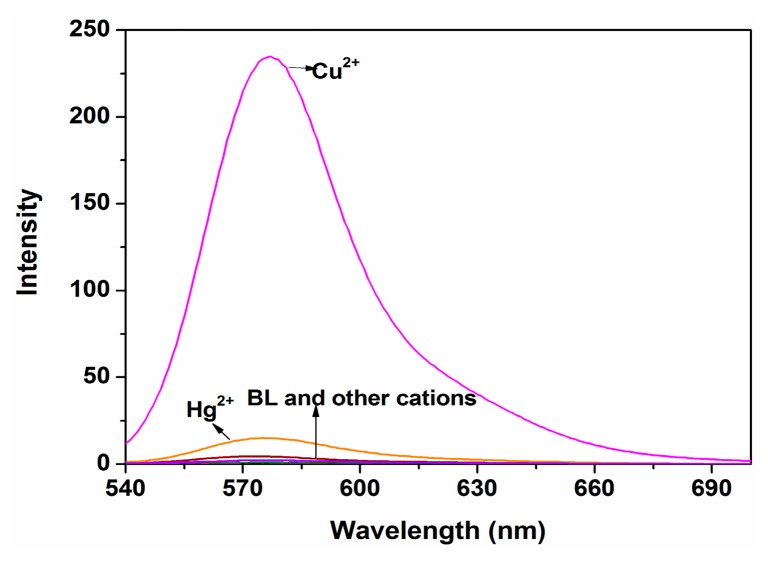
Fluorescence spectra of P (10 μM) with different metal ions (100 μM) in ethanol-water solution (9:1, v:v. 20 mM HEPES, pH 7.0).

To further evaluate the selectivity of probe P, the fluorescence spectra (ex = 520 nm) of P (10 μM) were investigated in ethanol-water solution (9:1, v:v, 20 mM HEPES, pH 7.0) with the addition of respective metal ions (100 μM) (Figure 3). Compared with other tested ions, only Cu^2+^ generated a significant “turn-on” fluorescence response of the monomeric peak at 577 nm with a fluorescence enhancement up to 200-fold, and Hg^2+^ had negligible interference. These results suggested that P had a higher selectivity toward Cu^2+^ than the other metal ions.

**Figure 4.**
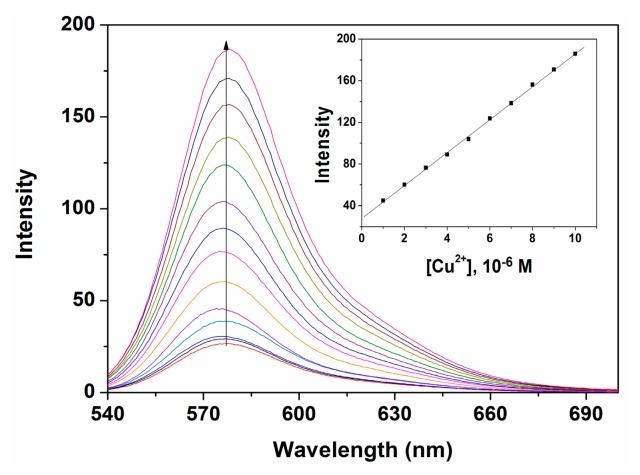
Fluorescence response of P (10 μM) with various concentrations of Cu^2+^ in ethanol-water solution (9:1, v:v. 20 mM HEPES, pH 7.0). Inset: the fluorescence of P (10 μM) as a function of Cu^2+^ concentrations (Em: 577 nm).

In order to investigate further the interaction of Cu^2+^ with P, a fluorescent titration experiment was carried out. The results showed that the fluorescence intensity of P was enhanced at 577 nm upon the addition of various amounts of Cu^2+^ in ethanol-water solution (9:1, v:v, 20 mM HEPES, pH 7.0), as depicted in Figure 4. Under the present conditions, when P was employed at the 10-μM level, the fluorescent intensity of P was proportional to the concentration of Cu^2+^ in the range from 1.0 × 10^−6^ to 1.0 × 10^−5^ M with a detection limit of 3.3 × 10^−7^ M Cu^2+^. This clearly demonstrated that chemosensor P could sensitively detect environmentally relevant levels of Cu^2+^.

### 3.4. Proposed Reaction Mechanism of P with Cu^2+^

**Figure 5.**
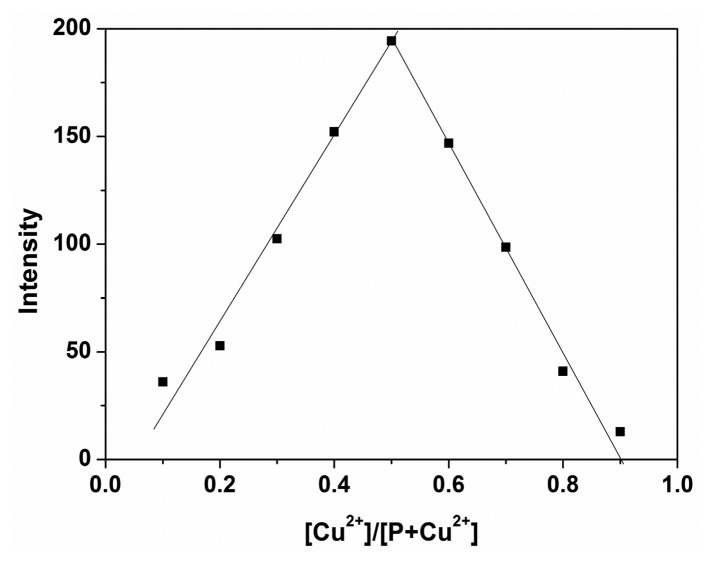
Job's plot of P with Cu^2+^. The total concentration of P and Cu^2+^ was kept at a fixed 50 μM.

**Scheme 2.**
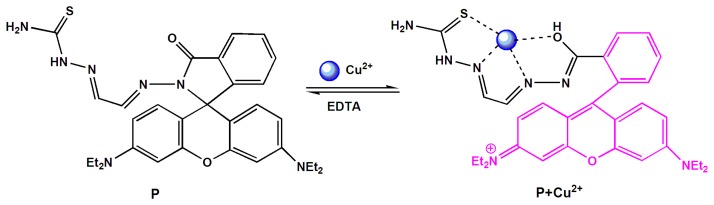
Proposed binding mode of P with Cu^2+^.

According to the obtained results above, the reaction mechanism is very likely due to the metal ion-induced ring opening of rhodamine spirolactam, rather than other possible reactions [12]. The Job's plot experiment was also explored to study the coordination mode of P with Cu^2+^, which confirmed a 1:1 stoichiometry for the P-Cu^2+^ complex and strongly supported the above conclusion (Figure 5). Thus, according to the obtained results, the coordination mode between P and Cu^2+^ was proposed as shown in Scheme 2.

**Figure 6.**
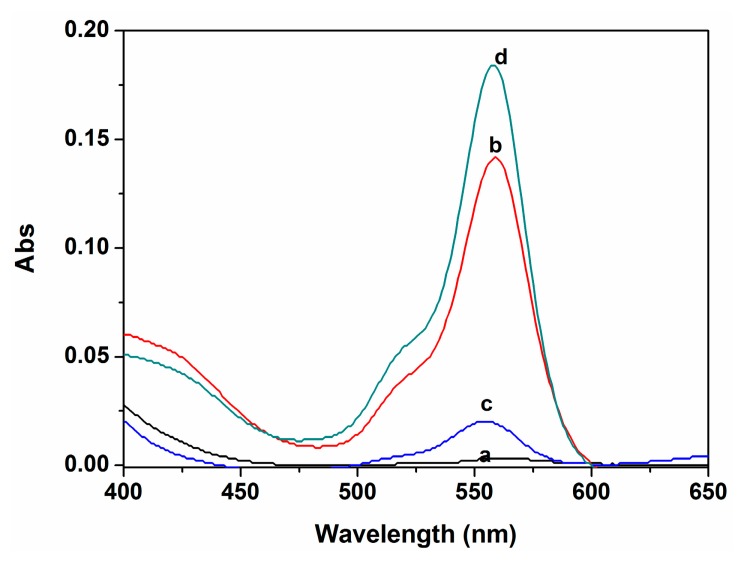
Absorption spectra in ethanol-water solution (9:1, v:v. 20 mM HEPES, pH 7.0): (a) P (10 μM); (b) P (10 μM) with Cu^2+^ (10 μM); (c) P (10 μM) with Cu^2+^ (10 μM) and then the addition of EDTA (50 μM); (d) P (10 μM) with Cu^2+^ (10 μM) and EDTA (50 μM) and then the addition of 0.1 mM Cu^2+^.

On the other hand, the response of P to Cu^2+^ was confirmed to be reversible by the EDTA titration. Upon addition of 50 μM EDTA to the mixture of P (10 μM) and Cu^2+^ (10 μM) in ethanol-water solution (9:1, v:v. 20 mM HEPES, pH 7.0), the color changed from pink to almost colorless, and a ∼93% fluorescent emission intensity of the system was quenched (Figure 6), which suggested that the EDTA replaced the receptor P to coordinate Cu^2+^. When Cu^2+^ was added to the system again, the signals were completely reproduced, and the colorless solution turned to pink. These findings indicated that P can be classified as a reversible chemosensor for Cu^2+^.

### 3.5. Preliminary Analytical Application

**Figure 7.**
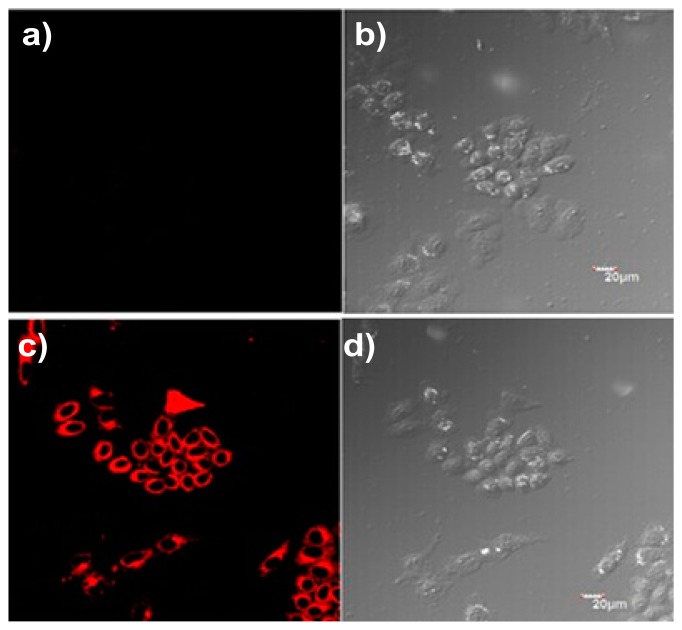
Confocal fluorescence and brightfield images of HepG2 cells. (**a**) Cells stained with 10 μM P for 30 min at 37 °C; (**b**) bright field image of cells shown in (a); (**c**) cells supplemented with 1 μM CuCl_2_ in the growth media for 30 min at 37 °C and then incubated with 10 μM P for 30 min at 37 °C; (**d**) brightfield image of cells shown in (c). (λ_ex_ = 559 nm).

To further demonstrate the practical applicability of the probe P, a cell imaging experiment was carried out to detect Cu^2+^ in living cells, and the fluorescence images of HepG2 cells were recorded before and after the addition of Cu^2+^ (Figure 7). The cells were supplemented with only P in the growth medium for 30 min at 37 °C, which led to very weak fluorescence, as determined by laser scanning confocal microscopy (ex = 559 nm) (Figure 7c). In contrast, when loaded with 1 μM CuCl_2_ for 30 min, a bright fluorescence was detected (Figure 7a). These results suggested that probe P can penetrate the cell membrane and might be used for detecting Cu^2+^ in living cells.

### 3.6. Method Performance Comparison

The performance of the proposed probe P was compared with the corresponding performance of some reported fluorescent probes for Cu^2+^ determination, as shown in Table 1. All of the fluorescent methods present good selectivity for Cu^2+^. The fluorescence quenching methods using rhodamine derivative exhibit a bad detection limit and are not applicable for living cells [13]. Most of the rhodamine derivatives possess good fluorescent properties [14–16], but some of them have more or less disadvantages, such as irreversibility [14] and complicated purification [14,15]. As for the two types of enhancement probes based on rhodamine derivative, dual-function detection for Cu^2+^ and ClO^−^ are realized [17], and a reversible response in living cells is exhibited [18]; however, low yields still restrict their further applications [17,18]. Our newly developed fluorescence enhancement method presents a number of attractive analytical features, such as a wide linear range, good reversibility and reproducibility, good selectivity and wide applicability. The fluorescence probe P based on rhodamine spirolactame derivative is easy to prepare at a low cost and can be used for routine analysis of ultra-trace levels of Cu^2+^ in living cells.

## 4. Conclusions

A new rhodamine B derivative was successfully characterized as a Cu^2+^-selective probe. The proposed probe has good selectivity towards Cu^2+^ compared with other common metal ions. Under optimized conditions, P exhibited a dynamic response range for Cu^2+^ from 1.0 × 10^−6^ to 1.0 × 10^−5^ M with a detection limit of 3.3 × 10^−7^ M Cu^2+^ in ethanol-water solution (9:1, v:v. 20 mM HEPES, pH 7.0).

## Supplementary Material



## Figures and Tables

**Table 1.** Performance comparison of various fluorescent probes for the Cu^2+^ ion.

**Fluorescence Modes**	**Fluorescence Reagents**	**Reproducibility**	**LOD (nM)**	**Testing Media**	**Applications**	**Remarks**	**Reference**
Enhancement λ_ex_/_em_ = 510/580 nm	Rhodamine derivative	Reversible	7	Water-methanol (2:8, v/v, pH 7.0, 20 mM HEPES)	HeLa cells	Yield (85%), simple synthetic route	[7]
Quenching λ_ex_/_em_ = 495/552 nm	Rhodamine derivative	NA	2600	Water-DMSO (1:1, v/v)	NA	Yield (52%)	[13]
Enhancement λ_ex_/_em_ = 530/575 nm	Rhodamine derivative	NA	10	Water-CH_3_CN (1:1, v/v, pH 7.1, 50 mM HEPES)	Waste water samples	Yield (60%), complicated purification	[14]
Enhancement λ_ex_/_em_ = 500/552 nm	Rhodamine derivative	Reversible	300	Water-ethanol (8:2, v/v, pH 7.1, Tris-HCl,)	River samples and HeLa cells	Yield (23%), complicated purification	[15]
Enhancement λ_ex_/_em_ = 510/580 nm	Rhodamine derivative	Reversible	3	Water-methanol (2:8, v/v, pH 7.0, 20 mM HEPES)	NA	Yield (80%), simple purification	[16]
Enhancement λex/em = 540/586 nm	Rhodamine derivative	NA	NA	Water-CH_3_CN (9:1, v/v, pH 7.0, 10 mM Tris-HCl)	NA	Yield (60%), dual-function chemosensor for Cu^2+^ and ClO^−^	[17]
Enhancement λ_ex_/_em_ = 495/552 nm	Rhodamine derivative	Reversible	NA	Water-CH_3_CN (1:1, v/v)	EJ cells	Yield (55%)	[18]
Enhancement λ_ex_/_em_ = 520/577 nm	Rhodamine derivative	Reversible	300	Water-ethanol (1:9, v/v, pH 7.0, 20 mM HEPES)	HepG2 cells	Yield (67.2%), simple synthetic route	This work
