# Supplementary material for: Synthesis of a Cu2+-Selective Probe Derived from Rhodamine and Its Application in Cell Imaging
Source: Sensors (Basel). 2014 Nov 12;14(11):21375–84. doi: 10.3390/s141121375 (PMC4279538; doi:10.3390/s141121375)
Supplement: Supplementary file 1 [file sensors-14-21375-s001.pdf]

OPEN ACCESS
sensors
ISSN 1424-8220
www.mdpi.com/journal/sensors

Supplementary Information

Synthesis of a Cu^{2+}-Selective Probe Derived from Rhodamine and Its Application in Cell Imaging. *Sensors* 2014, *14*, 21375-21384

Chunwei Yu †, Yingying Wen † and Jun Zhang *

Laboratory of Environmental Monitoring, School of Tropical and Laboratory Medicine, Hainan Medical University, Haikou 571199, China; E-Mails: yucw_1979@sina.com (C.Y.); wyy_418@163.com (Y.W.)

† These authors contributed equally to this work.

* Author to whom correspondence should be addressed; E-Mail: jun_zh1979@163.com; Tel.: +86-898-6697-3190; Fax: +86-898-6698-9173.

Supplementary Data

Figure S1. ESI-MS mass spectrum of P.

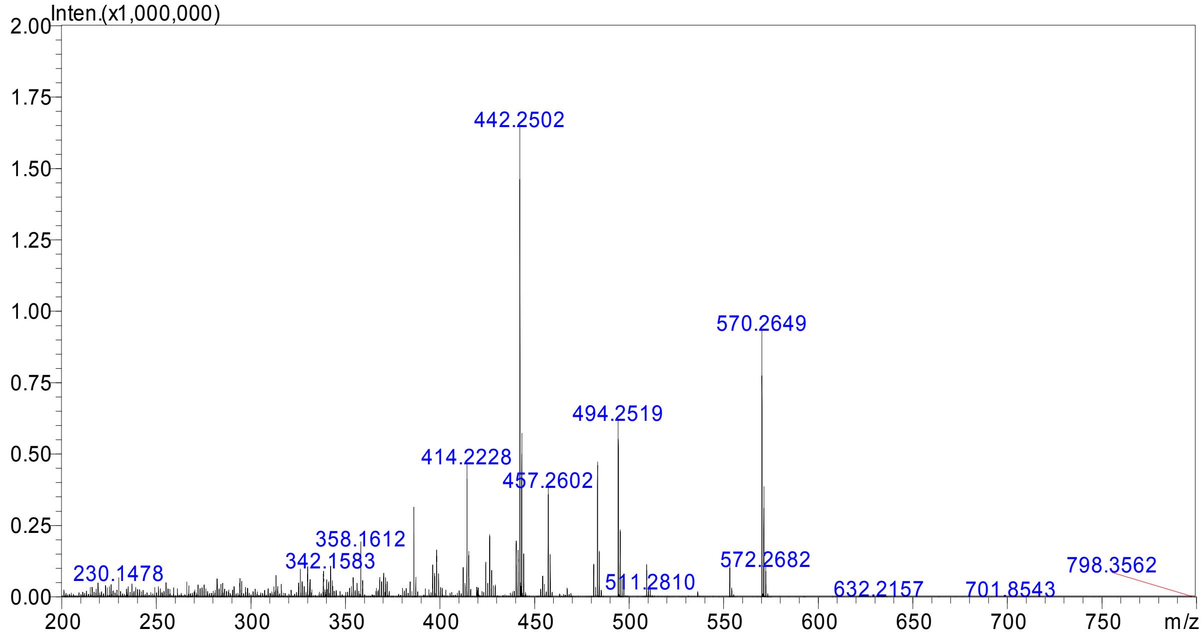

Figure S2. ^{1}H NMR spectrum of P.

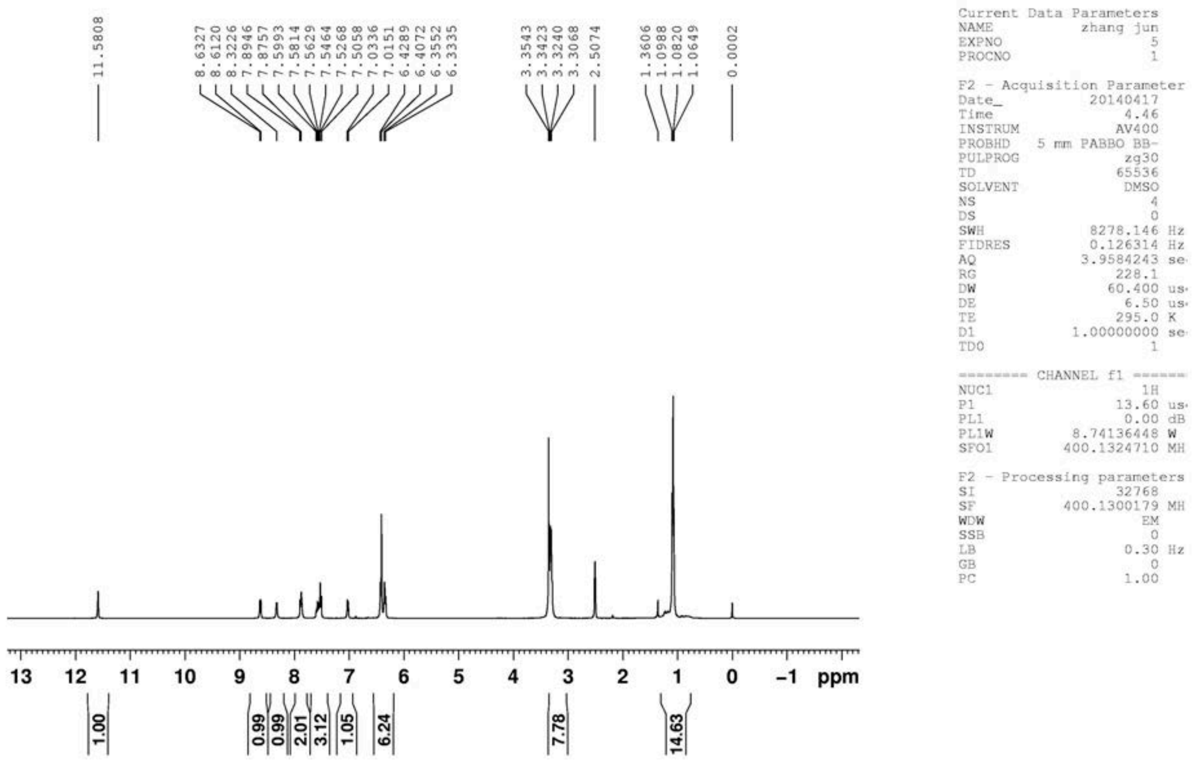

Figure S3. ^{13}C NMR spectrum of P.

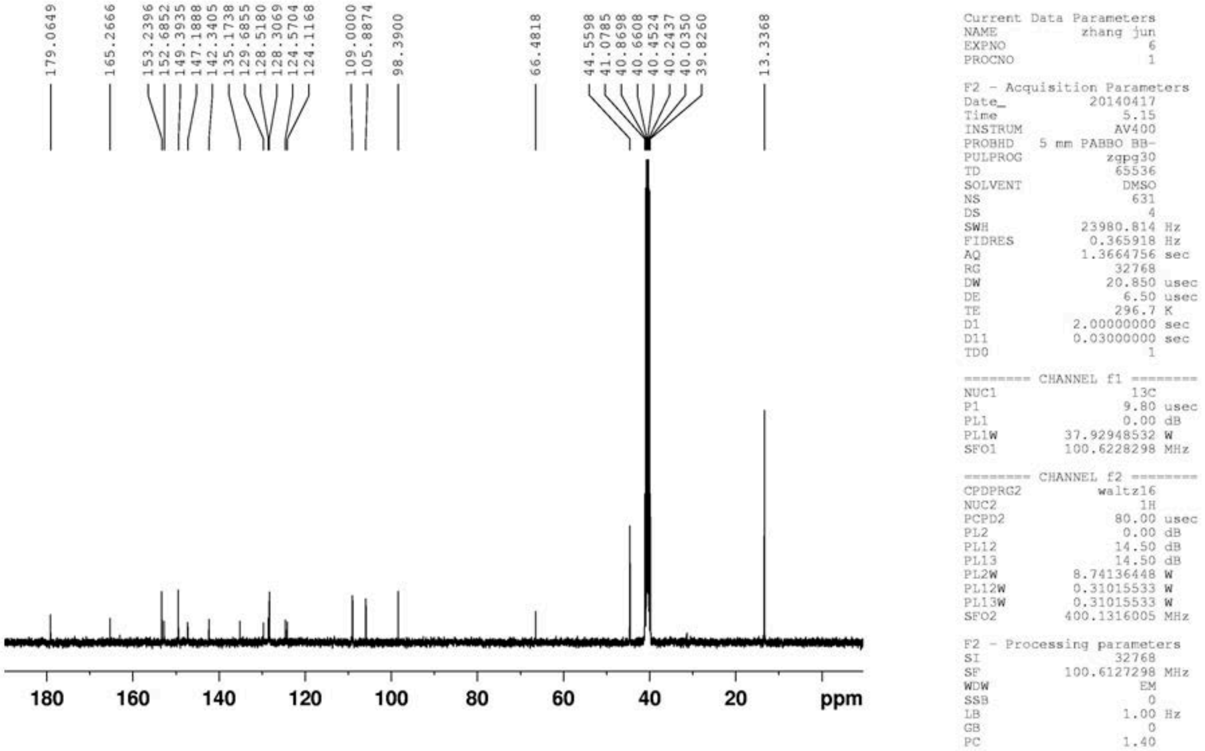

© 2014 by the authors; licensee MDPI, Basel, Switzerland. This article is an open access article distributed under the terms and conditions of the Creative Commons Attribution license (http://creativecommons.org/licenses/by/4.0/).
